# Cultural intelligence and acculturation among non-native Arabic learners: the impact of ‎learning apps

**DOI:** 10.12688/f1000research.149350.1

**Published:** 2024-05-17

**Authors:** Huda Abu-Qtaish

**Affiliations:** 1 Council on International Educational Exchange, Amman, Jordan

**Keywords:** Cultural Intelligence; Acculturation; Non-Native Learners; Arabic Language; ‎‎Learning Apps

## Abstract

**Background:**

Learning apps can be helpful to non-native language learners in learning Arabic, which includes speaking, writing, and speaking exercises. When learners become better in the language, they become more confident in interacting with the community, thus affecting their Cultural Intelligence (CQ) and Acculturation (AC). This study aimed to explore the relationship between the CQ and AC among non-native learners of Arabic. Additionally, the study aimed to investigate the potential impacts of learning apps and gender.

**Methods:**

This study used a correlational approach, involving a sample of 102 non-native Arabic language learners in Jordan. To assess these factors, this study used the Cultural Intelligence Scale and the Acculturation Survey.

**Results:**

The findings of this study revealed a positive correlation between the CQ and AC. Furthermore, the use of apps can provide CQ and AC levels. In addition, the study determined that gender did not play a significant role in influencing learners.

**Conclusion:**

the utilization of educational apps has been shown to enhance both CQ and AC. Thus, it is imperative to encourage learners to engage with these apps, as they foster cultural awareness, thereby facilitating the process of learning Arabic.

## Introduction

Learning the Arabic language as a second language has become a goal for many non-native learners from different countries, cultures, ages, and genders. Universities and institutes specialized in this field prepare curricula and programs for them with everything they need to learn the Arabic language as a second language considering that they will face some difficulties in adapting with the new culture or accepting the differences. Lecturers also recognize that this may be easy for some learners and difficult for others.

Foreign learners of the Arabic language when traveling to learn it in the country of the language, such as Jordan and Egypt, may suffer from difficulties in adapting to society because of their different cultures and lifestyles. Thus, foreign learners can adapt and achieve their goal of learning the Arabic language and interact with the environment and culture of the host country by acculturating with its people. This means that they have the ability to understand and interpret unfamiliar interactions from a cultural perspective ( Van Dyne, Ang, & Koh 2008).

Acculturation (AC) expresses the processes and stages that the learner of the second language goes through to adapt to the culture of native speakers ( Al-Osaily 2015). AC is one of the most essential elements for achieving true language competence. Language skills are not only mechanical, but also require social and cultural competence and communication skills with members of the target language community ( Boone 2021).

An individual’s possession of cultural intelligence (CQ) helps them to enhance their AC, as CQ expresses personal traits that focus on the behaviors and skills required to involve and interact with cultures. This helps the individual to become acquainted with the cultural environment of others and to engage, interact, and perform effectively in a manner commensurate with them, thus achieving an adequate understanding of the nature of the language and how to use it ( Thomas 2006). Aricat Karnowski, & Chib (2015) showed that people with high CQ who are culturally active are more in contact with members of society and develop AC than are individuals with low CQ who are fossilized.

Al-Muzaffar (2017) defines CQ as being able to deal with different environments and adapt to behavioral patterns in multiple cultures to engage with them easily. There can be no real communication in the target language without cultural efficacy; second language learners need to have an awareness and understanding of both the self-culture and the other’s culture to achieve success in communication in the context of “we.” Not everything that is said or written is always an indication of what is meant ( Parker 2016).

Coleman (2015) argued that studying abroad can provide huge opportunities for social interaction, and the depth of new social networks will lead to many results and outputs. The depth of learners’ acquaintance with new friends affects input, output, feedback, and the group of language functions that are practiced, and thus, pragmatic competence. In addition, greater contact with native speakers in their environment may affect other goals, such as academic, cultural, personal, professional, pleasure, and tourism. Therefore, increased communication with local communities led to greater gains.

Recently, educational apps have occupied an important position in education, including language teaching for foreigners ( Gangaiamaran & Pasupathi 2017). The advantage of learning apps is that they are easy to access anywhere and at all times ( Kuimova et al. 2018). Researchers have paid significant attention to technology, especially educational technology. Some research has focused on phones as assistants in language education ( Klimova 2019; Leis, Tohei, & Cooke 2015) and studies have found that the use of educational apps may have an effective role in teaching language to foreigners. This is because of their unique applications, such as activities, ease of use, and availability at any time ( Klimova 2019; Kukulska-Hulme 2016; Balula et al. 2015; Diána 2020).

There are many learning apps used by non-native Arabic learners, such as Duolingo, Learn Arabic Vocabulary, Memrise, Innovative 101, Pimsleur, Mondly, Teach me Arabic, and Rosetta Stone. In addition, there are many other apps available on Google Store and App Store that make learning languages easier and more effective ( Raed 2021).

The use of educational apps helps language learners to lessen their fears, practice, and feel linguistically efficient in communicating with native speakers ( Kacetl, J., & Klímová 2019; Klimova 2018), because educational apps contain various verbal and written lessons that help the individual practice correct pronunciation. When they use the correct words ( Chen 2016), this helps the individual in social engagement with others, and helps them exploit their CQ to interact and reach the AC that helps them achieve their goals ( Figueiredo et al. 2019; Vardakosta et al. 2023).

Many studies have investigated the effectiveness of using learning apps to learn languages for non-native learners ( Golonka et al. 2014; Hwang & Wang 2016; Liu et al. 2014; Hsu et al. 2014), but the focus was not on the impact of the use of learning apps in terms of cultural aspects, such as CQ and AC. Therefore, this study focuses on this effect.

### Research questions

1. Is there a statistically significant relationship between the CQ and AC among non-native Arabic learners?
2. Is there an impact of learning apps on the CQ and AC among non-native Arabic learners?
3. Are there statistically significant differences in the CQ and AC due to gender among non-native Arabic learners?

## Methods

### Study design

This study uses a correlational approach. One hundred and two non-native Arabic learners in Jordan were selected as subjects. All participants provided written informed permission with the scales. The research ethics committee authorized the institution’s study. Registration number: 2150/11/1/1. The date was 5/3/2023.

### Participants

The study sample consisted of 102 non-native Arabic learners in Jordan, who chose to participate voluntarily, aged between 19-24 years old. The participants studied at CIEE/Amman, the University of Jordan, and German Jordanian University. Informed consent was obtained verbally and written after participants were aware of the aims and subjects of this study.

### Data collection tools

*Cultural Intelligence Scale (CQS)*

The Cultural Intelligence Scale (CQ-S) was developed by ( Ahmad 2012; Ibrahim 2018), comprising 20 items and four dimensions (metacognitive, cognitive, motivational, and behavioral). A five-point scale were used, from 1 (‘strongly disagree’) to 4 (‘strongly agree’). The researcher elicited the validity by the discriminant evidence which ranged from 0.34 to 0.67. Also, the reliability was checked by Cronbach’s alpha test and retest the results was 0.91 and 0.88.

*Acculturation Survey (ACS)*

The Acculturation Survey (ACS) was developed by ( Palmer 2013; Dendrinos et al. 2014; Gbadamosi 2018), comprising 30 items and two dimensions (social and psychological), A five-point scale were used, from 1 (‘strongly disagree’) to 4 (‘strongly agree’). The researcher elicited the validity by the discriminant evidence which ranged from 0.32 and 0.77. Also, the reliability was checked by Cronbach’s alpha test and retest the results was 0.87 and 0.86.

### Data collection

Approval for this study was obtained from the University of Jordan Institutional Review Board. Also, the researcher took a written approval from the institutions and participants. The researcher collected the data by online instruments using Google forms. The period of applying the tools was 4 months during the semester.

### Data analysis

The researcher entered the data into SPSS data sheet, and used IBM SPSS v21 (IBM corp., Armonk, N.Y., USA) was used to analyze the data. Means and standard deviations were calculated, and Pearson’s correlation coefficient was used to determine relationships. An independent t-test was used to determine differences. All missing data are reported as missing.

## Results

### Levels of CQ and AC

The means and standard deviations were calculated to investigate the levels of CQ and AC. Table 1 presents the results.

**Table 1.** Means and standard deviations of CQ and AC.

Variables	Dimensions	Means	Std.	Level
CQ	metacognitive	4.19	0.57	High
	Cognitive	3.41	0.62	Moderate
	motivational	4.34	0.54	High
	behavioral	3.92	0.64	High
	Total CQ	3.92	0.46	High
AC	Social	3.65	0.42	Moderate
	Psychological	3.27	0.38	Moderate
	Total AC	3.50	0.28	Moderate

Table 1 shows that there is a high level of CQ among non-native learners of Arabic, with a mean of 3.91, and a moderate level of AC, with a mean of 3.50. Additionally, the CQ dimensions were high, whereas the cognitive dimension was moderate. The AC dimension was moderate.

### Relationships between CQ and AC

The Pearson correlation test was used to investigate the relationship between CQ and AC. Table 2 presents the results.

**Table 2.** The relationships between CQ and AC.

Variables	Total AC	Social	Psychological
Total CQ	.469 ^*^	.653 ^*^	-.216 *
Metacognitive	.378 ^*^	.485 ^*^	-.107
Cognitive	.422 ^*^	.563 ^*^	-.153
Motivational	.320 ^*^	.492 ^*^	-.223 *
Behavioral	.323 ^*^	.469 ^*^	-.181

* Correlation is significant at the 0.05 level.

Table 2 illustrates the correlation between the CQ and AC, with a Pearson coefficient of 0.469. Additionally, it revealed a connection between the dimensions of CQ and AC. However, no relationship was observed between the dimensions of the CQ and psychological dimension of AC.

### The impact of using learning apps on CQ and AC

To determine whether the use of learning apps affected CQ and AC, independent t-tests were used. The results of this study are presented in Table 3.

**Table 3.** Independent T-test results for the effect of using learning apps on CQ and AC.

Variable	Using apps	N	Means	Std.	T	Sig
CQ	No	61	3.75	0.40	5.18	0.00
	Yes	41	4.18	0.43		
AC	No	61	3.39	0.24	5.09	0.00
	Yes	41	3.65	0.27		

Table 3 demonstrates that app use can contribute to CQ and AC. The results revealed that learners who utilized learning apps achieved a higher mean score in both CQ and AC compared to learners who did not use them.

### The differences on CQ and AC due to gender

To determine whether there were differences in CQ and AC due to gender, an Independent T-test was used. The results of this study are presented in Table 4.

**Table 4.** Independent T-test results for the differences on CQ and AC due to gender.

Variable	Gender	N	Means	Std.	T	Sig
CQ	Male	30	3.93	0.44	0.06	0.95
	Female	72	3.92	0.47		
AC	Male	30	3.44	0.29	1.30	0.20
	Female	72	3.52	0.27		

Table 4 shows that there are no statistically significant differences on CQ and AC due to gender.

## Discussion

This research focuses on the use of learning apps among non-native language learners. Specifically, it focused on how learning apps affected the learning of Arabic languages, and the results found that it is affected. However, learning languages is not limited to vocabulary and reading; learners must know the culture and interact with the community. To this end, this research studied the relationship between CI and AC and the effect of learning apps on the participants. This study found that CI and AC had a positive effect on each other. CI refers to having many behavioral aspects and skills to help the learner communicate and get involved with the culture of the second language. This skill helps them achieve their goals of understanding the language and knowing how to use it in different situations. This was confirmed by Aricat et al. (2015), who mentioned that people with high CI will be more culturally active and become more involved with the community and develop their AC, and vice versa.

This study found that the use of learning apps increased CI and AC among non-native Arabic learners. With combined learning from apps as well as in the classroom, students show a greater understanding of the language. This, in turn, makes them more self-confident in their linguistic competence, which is the key to understanding culture. Learning apps contain verbal and written lessons that help learners break their fear of saying incorrect words. This helps learners get involved in the community, establish their CI in knowing others, and achieve their goal of becoming AC, leading them to learn Arabic more easily ( Klimova 2019; Diána 2020).

The study found that gender had no role in CI or AC among non-native Arabic learners. These results can be attributed to the fact that males and females face similar experiences when learning languages, such as being in a new country and culture shock ( Ellis 2015). Furthermore, both males and females have similar attitudes toward learning the Arabic language because they have left their homes to try learning it to fill their passion.

## Conclusion

This study revealed a significant correlation between CQ and AC among non-native learners, offering valuable insights into assisting them in adjusting to their new community. Furthermore, the utilization of educational apps has been shown to enhance both CQ and AC. Thus, it is imperative to encourage learners to engage with these apps, as they foster cultural awareness, thereby facilitating the process of learning Arabic. Notably, no discernible sex-based disparities were observed in terms of the CQ and AC.

### Limitations and future directions

This study had several limitations that warrant consideration. First, the research was conducted exclusively with a sample of non-native Arabic learners enrolled in Capital Amman, Jordan, during the year 2023. Consequently, the generalizability of the findings to broader populations and contexts may be limited. The study outcomes were based on participants’ responses to the CQ and AC scales.

To address these limitations and expand our understanding, we recommend several avenues for future research. First, it is advisable to conduct similar studies in diverse settings with more varied participant demographics to assess the robustness of the identified relationships. Second, the impact of learning apps on other variables that influence an individual’s ability to cope with a new community should be investigated. These variables include linguistic efficacy, social engagement, and psychological well-being.

Based on the insights gained from this study, it is advisable for universities and educational institutes to adopt a proactive stance by integrating additional online classes into their curriculum. This integration should involve the incorporation of educational apps as integral components of learning experience. By doing so, institutions can better facilitate learners’ cultural adaptation and language acquisition journey. This approach not only enhances the accessibility and convenience of learning but also promotes a more comprehensive understanding of the target culture and language.

Moreover, the researcher suggests that further investigation into the effects of learning apps on various variables that influence community coping is warranted. This would involve exploring how these apps impact factors beyond CQ and AC that are relevant to individuals’ abilities to navigate and integrate into a new community. By examining these additional variables, a more holistic perspective of the role of educational apps in fostering effective cultural adaptation can be attained. Future research will contribute to a deeper understanding of the multifaceted benefits of utilizing learning apps and their potential to enhance learners’ overall experience in a new cultural setting.

#### Ethics and consent

The University of Jordan Institutional Review Board, Jordan. Registration number: 2150/11/1/1. The date was 5/3/2023. All participants provided written informed consent with the scales.

## Data Availability

Figshare: Cultural Intelligence and Acculturation Among Non-Native Arabic Learners: The Impact of Learning Apps: **CQ & AC and apps.sav**, DOI: https://doi.org/10.6084/m9.figshare.25404190.v3 ( Abu-Qtaish, 2024a). This project contains the following underlying data:
• CQ & AC.sav **Reporting guidelines:** Figshare **:** CROSS check list: Checklist for Cultural Intelligence and Acculturation Among Non-Native Arabic Learners: The Impact of Learning Apps, at https://doi.org/10.6084/m9.figshare.25772271.v1 ( Abu-Qtaish, 2024b). Appendices: Scales for Cultural Intelligence and Acculturation Among Non-Native Arabic Learners: The Impact of Learning Apps, at https://doi.org/10.6084/m9.figshare.25772298.v1 ( Abu-Qtaish, 2024c). Data are available under the terms of the Creative Commons Zero (CC0).
