# Supplementary material for: Cultural intelligence and acculturation among non-native Arabic learners: the impact of ‎learning apps
Source: F1000Res. 2024 May 17;13:499. [Version 1] doi: 10.12688/f1000research.149350.1 (PMC11333874; doi:10.12688/f1000research.149350.1)
Supplement: Peer review history

# Reviewer report: Reviewer response for version 1

**DOI:** 10.5256/f1000research.163801.r310065

**Published:** 2024-09-04

**Authors:** Wan Ab Aziz Wan Daud

**Affiliations:** 1 Language Studies, University Of Malaysia, Kelantan, Bachok, Kelantan, Malaysia

Here are my comments as a reviewer on this article:

 This is an interesting and timely study examining the relationships between cultural intelligence (CQ), acculturation (AC), and the use of language learning apps among non-native Arabic learners. The research addresses an important topic given the increasing use of technology in language learning.

 Strengths:

 - Clear research questions and appropriate methodology to address them

 - Use of validated scales to measure CQ and AC 

 - Inclusion of both male and female participants

 - Consideration of the impact of learning apps, which is highly relevant

 - Statistical analyses appear appropriate for the research questions

 - Results are presented and discussed in relation to existing literature

 - Limitations and future directions are thoughtfully considered

 Suggestions for improvement:

 1. a more thorough literature review on CQ and AC in language learning contexts could benefit the introduction. This would help situate the current study more clearly.

 2. More details on participants' specific learning apps would be helpful. Were certain apps more commonly used? How frequently did participants use the apps on average?

 3. The discussion of gender differences (or lack thereof) could be expanded. Are there any theories or previous findings that would help explain the lack of gender differences observed?

 4. It may be worth considering potential mediating or moderating factors in the relationships between CQ, AC, and app use. For example, could factors like motivation or personality traits play a role?

 5. The practical implications of the findings could be elaborated on further. How specifically might language programs incorporate these findings to support learners?

 6. Some minor editing for grammar and clarity would improve readability in places.

 Overall, this is a solid study that meaningfully contributes to our understanding of cultural and technological factors in Arabic language learning. With some minor revisions, it would be suitable for getting indexed. The findings have clear relevance for language educators and program developers.

Is the work clearly and accurately presented and does it cite the current literature?

Yes

If applicable, is the statistical analysis and its interpretation appropriate?

Yes

Are all the source data underlying the results available to ensure full reproducibility?

Yes

Is the study design appropriate and is the work technically sound?

Yes

Are the conclusions drawn adequately supported by the results?

Yes

Are sufficient details of methods and analysis provided to allow replication by others?

Yes

Reviewer Expertise:

Arabic language: Instructional technology education

I confirm that I have read this submission and believe that I have an appropriate level of expertise to confirm that it is of an acceptable scientific standard.

# Reviewer report: Reviewer response for version 1

**DOI:** 10.5256/f1000research.163801.r310058

**Published:** 2024-08-19

**Authors:** Rahmah Binti Ahmad H. Osman

**Affiliations:** 1 International Islamic University Malaysia, Kuala Lumpur, Federal Territory of Kuala Lumpur, Malaysia

**COMPREHENSIVE EVALUATION OF THE JOURNAL ARTICLE TITLED "CULTURAL INTELLIGENCE AND ACCULTURATION AMONG NON-NATIVE ARABIC LEARNERS: THE IMPACT OF LEARNING APPS."**

**Title and Abstract**

- **Title**: The title is concise and appropriately reflects the study's focus on cultural intelligence (CQ), acculturation (AC), and the impact of learning apps among non-native Arabic learners.
- **Abstract**: The abstract provides a clear summary of the study, including the background, objectives, methods, results, and conclusions. However, the abstract could benefit from a clearer statement regarding the limitations and implications of the study.

**Introduction**

- **Relevance**: The introduction effectively introduces the topic and highlights the significance of CQ and AC in language learning. It places the study within the context of existing literature and clearly identifies the research gap.
- **Research Questions**: The research questions are well-formulated, focusing on the relationship between CQ and AC, the impact of learning apps, and the role of gender. These questions are appropriate and relevant to the study's objectives.

**Literature Review**

- **Comprehensiveness**: The literature review is comprehensive, covering various aspects of CQ, AC, and the role of learning apps in language acquisition. It effectively sets the stage for the research by discussing relevant theories and previous studies.
- **Critical Analysis**: While the literature review is thorough, it could benefit from more critical analysis of the studies cited. This would provide a deeper understanding of how the current study builds on or diverges from existing research.

**Methodology**

- **Study Design**: The study uses a correlational approach, which is appropriate for exploring the relationships between CQ, AC, and the use of learning apps. The study design is clearly explained, and the use of established scales for measuring CQ and AC adds to the study's credibility.
- **Sampling**: The sample size of 102 non-native Arabic learners is adequate for this type of study. The selection of participants from different universities in Jordan is justified, though more details on the sampling method would strengthen the study's validity.
- **Data Collection and Analysis**: The use of SPSS for data analysis is appropriate, and the statistical methods employed (e.g., Pearson's correlation, t-tests) are correctly applied. However, the article could provide more detail on the assumptions checked before performing these tests.

**Results**

- **Presentation**: The results are clearly presented in tables, making it easy to understand the findings. The study effectively reports the levels of CQ and AC, the relationship between them, and the impact of learning apps.
- **Interpretation**: The interpretation of results is consistent with the data presented. However, the discussion could benefit from linking the results more explicitly to the research questions and hypotheses.

**Discussion and Conclusion**

- **Discussion**: The discussion appropriately interprets the results in light of the existing literature. It highlights the significance of the findings and suggests practical implications for language learning and cultural adaptation.
- **Limitations**: The article acknowledges some limitations, such as the limited generalizability due to the specific sample. However, it would benefit from a more detailed discussion of other potential limitations, such as the use of self-reported data and the cross-sectional design.
- **Conclusion**: The conclusion succinctly summarizes the study's main findings and emphasizes the importance of learning apps in enhancing CQ and AC. It also suggests directions for future research, which adds value to the study.

**References**

- **Consistency**: The reference list is generally well-prepared, but there is an inconsistency in citation styles (e.g., APA vs. Vancouver). This should be corrected for uniformity.
- **Coverage**: The references cited are relevant and current, covering a broad range of sources related to the study's topic. However, the inclusion of more recent studies on the impact of technology on language learning could strengthen the literature review.

**Ethical Considerations**

- **Ethical Approval**: The study mentions that ethical approval was obtained from the University of Jordan Institutional Review Board, and informed consent was obtained from participants. This adherence to ethical standards is commendable.

**Overall Assessment**

- **Strengths**: The article is well-structured and covers an important topic in language learning. The use of established scales and appropriate statistical methods adds to its credibility.
- **Areas for Improvement**: The article could benefit from more critical analysis in the literature review, a more detailed discussion of limitations, and consistency in referencing style. Additionally, the abstract could better highlight the study's limitations and implications.

**Recommendation**

- **Indexing**: The article is of good quality and contributes valuable insights into the field of language learning. It is recommended for indexing after addressing the minor issues identified, particularly in the references and discussion sections.

This evaluation adheres to conventional standards and provides a balanced assessment of the article's strengths and areas for improvement.

Is the work clearly and accurately presented and does it cite the current literature?

Yes

If applicable, is the statistical analysis and its interpretation appropriate?

Yes

Are all the source data underlying the results available to ensure full reproducibility?

Yes

Is the study design appropriate and is the work technically sound?

Yes

Are the conclusions drawn adequately supported by the results?

Yes

Are sufficient details of methods and analysis provided to allow replication by others?

Yes

Reviewer Expertise:

Technologies and the teaching of Arabic Studies

I confirm that I have read this submission and believe that I have an appropriate level of expertise to confirm that it is of an acceptable scientific standard.

# Reviewer report: Reviewer response for version 1

**DOI:** 10.5256/f1000research.163801.r284126

**Published:** 2024-06-19

**Authors:** Nadia Mustafa Alassaf

**Affiliations:** 1 Head of teaching Arabic for foreign department, The University of Jordan, Amman, Amman Governorate, Jordan

This research studied the learning apps used by non-native Arabic learners, especially after the Covid-19 pandemic. It studied the effect of using apps on acculturation and cultural intelligence which are important variables for them to learn Arabic and to get high scores in the subjects. These to variables are social variables that can help them to cope and interact with the native speakers in Jordan and prevent them from facing a lot of problems while living in the Arabian country.

 This research is designed in an appropriate manner and divided in a correct scientific manner. While reading the research, I found that the researcher was aware and knowledgeable about the subject of her study. The researcher was very precise in defining the questions of her study, as they were not random, and thus this was reflected in her results. The results of her study may be a starting point for other scientific research. It is worth noting that the researcher has addressed in her research a new aspect that has not been focused on much in scientific research that addressed the same topic. Previous research addressed the impact of applications on foreign students in terms of learning and acquiring the language, not in terms of culture, especially acquiring the culture of the society in which they live outside their home country and integrating into it. Based on the above, we strongly recommend indexing this research to benefit from it in the field of teaching Arabic as a second language.

 This research can be improved by doing these issues:

 1) The reference “Aricat, R. G., Karnowski, V., & Chib, A. (2015)” was written according to APA7 style while other references were written according to Vancouver style.

 2) On the levels results I prefer to write the total level and the dimensions level together not separated.

 3) I read research about techno wellness and may be helpful for you to improve discussion, it’s means how to use technology to improve psychological wellness and include academic and social wellness.

Is the work clearly and accurately presented and does it cite the current literature?

Yes

If applicable, is the statistical analysis and its interpretation appropriate?

Yes

Are all the source data underlying the results available to ensure full reproducibility?

Yes

Is the study design appropriate and is the work technically sound?

Yes

Are the conclusions drawn adequately supported by the results?

Yes

Are sufficient details of methods and analysis provided to allow replication by others?

Yes

Reviewer Expertise:

Specialized in teaching Arabic as a second language and published many scientific research and articles in peer-reviewed journals and journals within Scopus.

I confirm that I have read this submission and believe that I have an appropriate level of expertise to confirm that it is of an acceptable scientific standard.
